# Prognostic value of ^18^F-FDG PET/CT parameters and histopathologic variables in head and neck cancer

**DOI:** 10.1016/j.bjorl.2019.10.014

**Published:** 2019-12-10

**Authors:** Hale Aslan, Gul Cekin, Ercan Pinar, Mustafa Yazir, Abdulkadir Imre, Murat Songu, Akif Islek, Ibrahim Aladag, Ismail Semih Oncel

**Affiliations:** a Katip Celebi University School of Medicine, Department of Otolaryngology Head and Neck Surgery, Izmir, Turkey; b Katip Celebi University School of Medicine, Department of Nuclear Medicine, Izmir, Turkey; c Mardin Nusaybin State Hospital, Mardin, Turkey

**Keywords:** Head and neck cancer ^18^F-FDG PET/CT, Prognosis, Metabolic parameters

## Abstract

**Introduction:**

^18^F-fluorodeoxyglucose positron emission tomography/computed tomography parameters such as; maximum standardized uptake values, standard metabolic tumor volume and otal lesion glycosis are important prognostic biomarkers in cancers.

**Objective:**

To investigate the prognostic value of these parameters in patients with head and neck cancers.

**Methods:**

We performed a retrospective study including 47 patients with head and neck cancer who underwent^18^F-fluorodeoxyglucose positron emission tomography/computed tomography prior to treatment. Standard metabolic tumor volume, otal lesion glycosis and standardized uptake were measured for each patient. The prognostic value of quantitative ^18^F-fluorodeoxyglucose positron emission tomography/computed tomography parameters and clinicopathologic variables on disease free survival and overall survival were analyzed.

**Results:**

The median (range) standard metabolic tumor volume and otal lesion glycosis and standardized uptake were 7.63 cm^3^ (0.6–34.3), 68.9 g (2.58–524.5 g), 13.89 (4.89–33.03 g/mL), respectively. Lymph node metastases and tumour differentiation were significant variables for disease free survival and overall survival, however, all ^18^F-fluorodeoxyglucose positron emission tomography/computed tomography parameters were not associated with disease- free survival and overall survival.

**Conclusion:**

Pretreatment quantities positron emission tomography parameters did not predict survival in head and neck cancer.

## Introduction

Head and neck carcinomas are an important cause of mortality in humans. Head and neck carcinomas are clinically heterogeneous entities that show disparities in natural course or clinical behaviour depends on the tumor location and histopathologic variables such as, tumor size, stage, lymph node metastases, surgical margin and lymphovascular invasion.^1^, ^2^ However, despite careful evaluation of these factors, it is not possible to reliably predict the outcome of treatment inpatients.

^18^F-FDG PET/CT is based on tumour glucose metabolism and serves as a marker of tumour metabolic activity in terms of cell viability and proliferation. It has been successfully applied in pre-treatment staging, treatment response assessment and post treatment follow-up. It is superior to computed tomography and magnetic resonance imaging in the detection of carcinomas of unknown primary, cervical lymph node or distant metastasis.^3^, ^4^

Recently, ^18^F-FDG Metabolic Tumor Volume (MTV) and Total Lesion Glycolysis (TLG), combining the tumour volume and metabolic activity of the entire tumur have been introduced as prognostic biomarkers in various solid malignancies, however their prognostic values are not well established.^5^, ^6^ The value of MTV was already identified in patients with HNSCC who received radiotherapy.^7^ In particular, the question of which is the better parameter to predict outcome remains unresolved.

We performed this retrospective study to determine the yield of pretreatment quantitative ^18^F-FDG PET/CT parameters and histopathologic variables for the prediction of OS, DFS and lymph node metastasis.

## Methods

This retrospective study was approved by the institutional review board (19-12-2018/423).

### Patients

The population of this retrospective study consisted of patients with locally advanced head and neck squamous cell carcinoma involving oropharnyx, hypopharynx, oral cavity and larynx who had FDG PET/CT for initial staging between January 2015 and June 2016. Exclusion criteria were non- squamous cell carcinoma histology, previous treatment, and evidence of distant metastasis. Cancers originating in the salivary gland, thyroid and paranasal sinuses were also excluded. 47 patients were included in this study. All patients underwent surgical resection of primary tumour with cervical neck dissection, followed by radiotherapy with or without concurrent chemotherapy. Indications for postoperative adjuvant therapy relied on postoperative pathologic features. All patients were followed at least two years.

### F-FDG PET/CT

Patients with glucose level lower than 200 mg/dL fasted for 6 h prior to intravenous infusion of 0.1 mCi/kg ^18^F-FDG. PET/CT was performed on GE Discovery 710. CT (50 mAs, 120 kV, 3.75 mm section thickness) was performed without intravenous contrast administration. PET scan was performed immediately after CT and whole body images from skull base to upper thigh were scanned on average 7–8 bed positions (2 min per bed position).

### Image analysis

Volumetric region of interest were placed over areas of abnormal ^18^F-FDG uptake. Standardized uptake values (SUV_max_ and SUV_mean_), Metabolic Tumor Volume (MTV) and Total Lesion Glycolysis (TLG) of primary tumor and neck lymph nodes were evaluated for each patient. A threshold of 42 % of maximum signal intensity was used to delineate the MTV. TLG was calculated as the product of lesion means standardized uptake value and MTV. FDG uptake was defined to be positive qualitatively when a focal FDG uptake was higher than the normal biodistribution of background FDG activity. After correlating abnormal CT findings, increased focal FDG uptake was accepted pathological diseases. When the positive lymph nodes were evaluated, its size and metabolic activity were described. In the presence of more than one lymph node, anatomically the largest one and the most active lymph nodes were described.

### Statistical analysis

All statistical analysis was performed using the SPSS windows 15.0 software (SPSS, Chicago, Illionis). Univariate analysis was used to identify clinical factors and imaging parameters predictive of Disease-Free Survival (DFS) and Overall Survival (OS) by using the ×2 or Fisher’s exact test. We determined the cut-off values for various FDG-uptake parameters (SUV_max_, MTV, TLG). Disease free survival and overall survival curves were calculated using the Kaplan-Meier method. The log-rank test was used to compare survival rates according to imaging parameters. In the univariate analysis, all patients were separated into 2 groups based on the SUV_max_, MTV and TLG. The relationships between the two groups of each FDG-uptake parameters with survival and lymph node metastases were compared using χ^2^ test. A value of *p* less than 0.05 was considered significant.

## Results

### Patient characteristics

47 patients were included in this study. The study group was composed of 40 male and 7 female patients with the mean age of 63 years (range 43–89 years). All patients included in this study were diagnosed with locally HNSCC, including 16 TNM stage IV, 15 Stage III and 16 Stage II. The larynx was the most common site of primary tumour, observed in 31 patients (65.9 %). Poor differentiation were observed in 20 patients (42.5 %). The tumour progression was identified in 8 patients (17 %): in a local site in 2 (4 %) patients, in a regional site in 3 patients and distant sites in 3 patients in the follow-up. 39 patients were alive without disease for 3 years.

**Figure 1:**
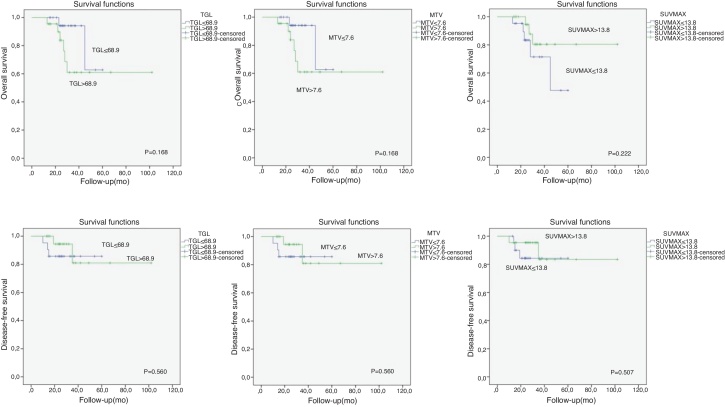
Survey analysis of ^18^F-FDG PET/CT metabolic parameters.

The median MTV, TLG and SUV_max_ were 7.63 cm^3^ (0.6–34.3), 68.9 g (2.58–524.5 g), 13.89 (4.89–33.03 g/mL), respectively. There was no significant relationship between PET parameters and survival. Only lateral lymph node metastases and tumor differentiation reached significance (Table 1). SUV_max_, MTV and TLG values did not reach significance for patients with lymph node metastasis. TNM stage and perineural or perivascular invasion were significantly associated wilt lymph node metastases (Table 2). From the ROC curve analysis, the SUV_max_, MTV and TLG cut-off values for patients with survival relationship were shown in Fig. 1.

**Table 1:** Analysis of PET/CT parameters and clinicopathologic parameters in relation to DFS and OS.

Variables	3 year DFS (%)	p-value	3 year OS (%)	*p*-value
Lateral LN		0.051		0.044
Metastatic	68		56	
Reactive	82		78	
TNM		0.0473		0.291
Stage 1‒2	92		92	
Stage 3‒4	79		71	
Differantiation		00.5		0.012
Poor dif	95		88	
Mild dif	83		82	
Well dif	67		47	
Tm size		0.769		0.477
0‒2 cm	80		100	
2‒4 cm	82		73	
Up to 4 cm	90		80	
PN‒PV inv		0.187		0.480
PNor PV(+)	44		86	
PN or PV(-)	91		73	
Suv max		0.507		0.222
≥ Median level	84		80	
≤ Median level	84		71	
TLG		0.560		0.168
≥ Median level	81		61	
≤ Median level	86		94	
MTV		0.380		0.264
≥ Median level	75		51	
≤ Median level	78		84	

Assessed using the two-sided Fisher’s exact test or the χ^2^ test.

PN, Perineural Invasion; PV, Perivascular Invasion.

**Table 2:** Relationship between PET parameters and histopathologic parameters and lymph node metastases.

Variables	Lateral lymph node metastasis		*p*-value
	Positive	Negative	
TNM			0.005
Stage 1‒2	3	10	
Stage 3‒4	16	10	
Differentiation			0.658
Poor dif.	10	7	
Mode dif.	5	7	
Well dif.	4	6	
pT stage			0.0585
T1	1	2	
T2	11	14	
T3‒T4	7	4	
PN-PV inv			0.003
PNor PV(+)	8	2	
PN or PV(-)	7	22	
Suv_max_			0.307
≥ Median level	11	11	
≤ Median level	8	9	
TLG			0.721
≥ Median level	13	9	
≤ Median level	6	11	
MTV			0.759
≥ Median level	12	10	
≤ Median level	7	10	

Assessed using the two-sided Fisher’s exact test or the χ^2^test.

PN, Perineural Invasion; PV, Perivascular Invasion.

## Discussion

FDG PET/CT simultaneously providing anatomical and functional information is useful for the detection of primary tumor metastases, synchronous primary tumor, RT planning, assessment of treatment response and surveillance for tumor recurrence and metastases after treatment.^1^ Functional imaging of FDG PET/CT can provide metabolic information on malignant tissues and make it possible to reflect the tumor burden more accurately. Various F-FDG PET parameters have been investigated in solid tumours, MTV, which is a measure of the volume of the tumour displaying F-FDG uptake and quantifies the overall tumour burden, is a better predictor of outcome than SUV_max_. TLG have been developed to measure metabolic activity in an entire tumor mass.^8^, ^9^, ^10^ Therefore, volume-based parameters such as MTV and TLG may reflect the metabolic burden of the active tumour more accurately than SUV_max_.

Many studies have shown that elevated MTV,TLG and SUV_max_ values is associated with worse prognosis.^7^, ^11^, ^12^ Pretreatment SUV_max_ has been used to evaluate the aggressiveness of disease, response to therapy, early detection of recurrence and outcome, however, previous studies and the present study showed that elevated MTV,TLG and SUV_max_ values were not significantly associated with lymph node metastasis and survival.^13^, ^14^ The risk of lymph node metastasis and decreased OS and DFS was not higher in patients with high MTV, TLG and SUV_max_ values. High FDG uptake correlated with advanced pT and pN stages. However, the SUV_max_ of primary tumor, including metastatic lymph nodes was not confirmed as a significant factor predicting clinical outcome in HNSCC patients. In our study, lower SUV_max_ values did not demonstrate higher rates of OS and DFS with a statistical significance.

TNM stage, histopathologic parameters are important prognostic factors in HNSCC.^1^ Pathologic T and N stages were significant predictive factors of clinical outcome in the study by Le Tourneau et al.^15^ On the contrary, other studies showed different results and pT and pN stages did not predict treatment outcome and survival.^16^, ^17^ In this study, stage and perineural or perivascular invasion was significantly associated with lymph node metastases. Only lymph node metastases and tumour differentiation was associated with decreased OS and DFS.^7^

Our study had several limitations, including its retrospective design, small number of patients and lack of 5 years OS and DFS. Head and neck cancers show a disparity in natural course or clinical behavior depending on primary location and histopathologic properties.

## Conclusion

In conclusion, pretreatment MTV, TLG and SUV_max_ did not predict poor prognosis in our study. Only tumour differentiation and lymph node metastases were associated with lower survival. Perineural and perivascular invasion and TNM stage were the predictors of lymph node metastases. Additional prospective studies with a larger number of patients are needed to validate the prognostic predictors of these functional biomarkers derived from PET/CT.

## Conflicts of interest

The authors declare no conflicts of interest.
